# The Influence of Curing Systems on the Cure Characteristics and Physical Properties of Styrene–Butadiene Elastomer

**DOI:** 10.3390/ma13235329

**Published:** 2020-11-25

**Authors:** Magdalena Maciejewska, Monika Siwek

**Affiliations:** Institute of Polymer and Dye Technology, Lodz University of Technology, Stefanowskiego Street 12/16, 90-924 Lodz, Poland; 212599@edu.p.lodz.pl

**Keywords:** curing system, Activ8, elastomer, vulcanization, zinc oxide, SBR

## Abstract

The goal of this work is to study the influence of different curing systems on the cure characteristics and performance of styrene–butadiene elastomer (SBR) filled with carbon black or nanosized silica. A multifunctional additive for rubber compounds, namely Activ8, was applied as an additional activator and accelerator to increase the efficiency of sulfur vulcanization and to reduce the content of zinc oxide elastomers cured in the presence of 2-mercaptobenzothizole or 1,3-diphenylguanidine as a primary accelerator. The influence of the curing system composition on the crosslink density and physical properties of SBR vulcanizates, such as mechanical properties, thermal stability, and resistance to thermo-oxidative aging, is also reported. Activ8 effectively supports the vulcanization of SBR compounds, especially filled with nanosized silica. It reduces the optimal vulcanization time of SBR compounds and increases the crosslink density of the vulcanizates. Moreover, vulcanizates with Activ8 exhibit higher tensile strength and better damping properties than elastomer with zinc oxide. Activ8 allows the amount of ZnO to be reduced by 40% without detrimental effects on the crosslink density and mechanical performance compared to the vulcanizates conventionally crosslinked with ZnO. This is an important ecological goal since ZnO is classified as being toxic to aquatic species.

## 1. Introduction

Vulcanization is one of the oldest and undoubtedly most important processes in elastomer technology, leading to the transformation of a plastic rubber compound into a highly elastic finished product—that is, a vulcanizate, characterized by specific functional properties. From a chemical point of view, this process consists of the creation of a three-dimensional spatial network in the vulcanizate, in which rubber macromolecules are linked by crosslinks formed during crosslinking reactions [1]. In addition to curing agents, other auxiliaries are commonly used to ensure the proper course of the vulcanization process. The most important ones include vulcanization accelerators and activators, vulcanization retarders and inhibitors, and crosslinking coagents [2]. Since vulcanization has been a known process for over 180 years, many different curing systems have been developed across this period to vulcanize rubber compounds, the most important of which are based on the use of sulfur, peroxides, and metal oxides [2]. The choice of a proper curing system results mainly from the chemical nature of the rubber (e.g., the degree of unsaturation) and the presence of functional groups suitable for crosslinking reactions (e.g., carboxyl and chlorosulfone). The most widely used curing agents in the rubber industry are elemental sulfur for rubbers with some degree of unsaturation [3] and peroxides for saturated copolymers, e.g., ethylene–propylene copolymer [4].

The reaction of rubber with sulfur is slow even at high temperatures. Therefore, in recent years, efforts have been made to accelerate the vulcanization process. Nowadays, accelerators and activators are crucial ingredients in sulfur curing systems, which govern the main parameters of the vulcanization, such as temperature and time, as well as the safety of rubber compound manufacturing. In addition to the accelerating effect, applying accelerators and activators increases the crosslink density and improves the efficiency of sulfur consumption to form crosslinks, consequently reducing the amount of sulfur in the rubber products. Depending on the chemical structure and influence on the cure rate, accelerators are divided into four classes: slow (guanidines), medium fast (thiazoles and sulfenamides), very fast (thiurams) and ultra-accelerators (dithiocarbamates and xanthates). Some of them, e.g., sulfenamides, provide a delayed action to the vulcanization, ensuring a good balance between the scorch safety and the cure rate. Therefore, regarding the manufacturing of rubber composites and the properties of the final elastomers, thiazoles and sulfenamides are preferable due to the long induction period of the vulcanization and the fast main crosslinking process [5].

Apart from the course and rate of the vulcanization, the composition of the curing system has been reported to affect the final mechanical properties of vulcanizates, including their thermal stability and resistance to thermal aging [6,7]. These properties are strongly dependent on the crosslink structure in the created elastomer network [8]. The crosslink structure is basically determined by the degree of crosslinking or the crosslink density, as well as the polysulfidity of the crosslinks in the network structure of the elastomer. These, in turn, depend mainly on the sulfur content and sulfur/accelerator ratio in the curing system used. No less important is the type of accelerator, which affects the efficiency of sulfur consumption during vulcanization and, consequently, the crosslink density of the final vulcanizate. Thus, regarding the sulfur/accelerator (S/A) ratio, curing systems are grouped into conventional (CV), semi-efficient (SEV), and efficient (EV). The S/A ratio in CV systems is higher than in the other systems, resulting in longer predominantly polysulfidic crosslinks. The presence of polysulfidic crosslinks in the elastomer network improves the dynamic fatigue resistance, but deteriorates the thermal stability and resistance to thermal aging of the cured material. In addition, CV vulcanizates usually exhibit higher crosslink density than rubber compounds cured with an EV system. Meanwhile, SEV systems consist of an equivalent amount of sulfur and accelerator, providing optimal mechanical performance and thermal behavior of the elastomer. In contrast, EV systems are characterized by the lowest S/A ratio, resulting in an elastomer network with predominantly mono- and disulfidic crosslinks, which provide better thermal aging resistance, higher stiffness of the material, and low ability for stress relaxation [6]. According to Coran 2003, the S/A ratio has a significant influence on the modifications of the main chain of the rubber during vulcanization [3]. For example, a higher amount of the accelerator with respect to the sulfur content increases the number of pendant groups formed during crosslinking reactions, which dangle from the rubber molecular chains. Such modifications in the structure of the elastomer network affect the properties of the final vulcanizates. The best mechanical performance is demonstrated by vulcanizates with long-chain polysulfidic crosslinks in the elastomer network, but with little modification of the main chain of the rubber [3].

Most importantly, when analyzing the literature on vulcanization from the last few years, it can be seen that most of the research focuses only on the influence of the composition of the curing system in terms of the S/A ratio on the properties of elastomer composites. However, the type of accelerator used is no less important, especially when several accelerators are used simultaneously, which may have a synergistic effect and interact with each other. For instance, Marković et al., 2009 studied the influence of the accelerator type on the cure characteristics and performance of sulfur-cured rubber blends consisting of natural rubber (NR) and chlorosulfonated polyethylene (CSM) [9]. N-cyclohexyl-2-benzothiazole sulfenamide (CBS), tetramethylthiuram disulfide (TMTD), and 2-mercaptobenzothiazole (MBT) were used as vulcanization accelerators. The type of accelerator used not only affected the cure characteristics of the rubber blends, but also had a significant impact on the mechanical performance of the vulcanizates. The highest cure rate and torque increment during the vulcanization were indicated for the rubber blends cured with TMTD, whereas the lowest were for the MBT-containing composites. Regarding the mechanical properties, the highest modulus at 300% elongation, tensile strength, and hardness were shown by the NR/CSM blends cured with TMTD. In contrast, the MBT-containing vulcanizates exhibited the worst performance, probably due to the lowest crosslink density.

Azar et al., 2017 studied the influence of the types of accelerator on the network structure and properties of NR/chloroprene rubber blends. A strong interdependence was reported between the type of accelerator and the stress relaxation behavior of the vulcanizates, as well as their network structure, crosslink density, aging resistance, and, finally, decomposition energy [10].

Gobbi et al., 2020 compared the use of TMTD, tetrabenzylthiuram disulfide (TBzTD), and zinc dibenzyldithiocarbamate (ZBEC) as accelerators in the sulfur vulcanization of isobutylene–isoprene rubber compounds. The highest maximum torque during the rheometric measurements, as well as the highest hardness and modulus at 100% and 300% relative elongations, were achieved for the elastomers cured with TMTD. This was due to the highest crosslink density of the TMTD-containing vulcanizates, since TMTD acts as a sulfur donor, increasing the content of sulfur in the curing system [11].

Alam et al., 2012 studied the influence of zinc dithiocarbamates and thiazole-based accelerators on the vulcanization of NR compounds [12]. It was reported that the gradual substitution of zinc dimethyldithiocarbamate (ZDMC) by dibenzothiazyl disulfide (MBTS) resulted in a synergistic activity of these accelerators concerning the torque increment, which could be observed as the improved efficiency of the curing process. The synergistic effect of ZDMC and MBTS accelerators was also noticed for the stresses at different relative elongations and the tensile strength of the vulcanizates. The scorch time, optimal vulcanization time, and elongation at break of the NR composites were within the values obtained for the vulcanizates containing ZDMC or MBTS separately. The synergistic activity of the ZDMC and MBTS accelerators with regard to the cure characteristics and mechanical behavior of the vulcanizates was also noticeable for the curing systems composed of ZDMC and CBS accelerators. The synergistic activity of the accelerators was reported to result from the reclamation of the zinc dithiocarbamates (ZDCs) in the presence of other additives.

Regarding industrial applications, thiazoles, sulfenamides, and dithiocarbamates were the last introduced class of accelerator of great commercial importance. It should be noted that the development of advanced technologies forces the industry to manufacture rubber products with more sophisticated commercial properties. Therefore, it becomes necessary to add more and more components to rubber compounds, which increases the complexity of their recipes and affects their manufacturing process. Manufacturers cannot rely only on the components of those curing systems that have been used for years. Instead, it is more reasonable to look for new substances that could be an alternative to traditionally used additives, capable of simultaneously providing rubber products with new, additional properties. Therefore, in this work, a new additive for rubber compounds, namely, Activ8, was applied as an additional activator and accelerator for the sulfur vulcanization of styrene–butadiene elastomer (SBR) in order to increase the efficiency of the vulcanization and to reduce the content of zinc oxide in rubber compounds cured in the presence of MBT or 1,3-diphenylguanidine (DPG) as a primary accelerator. We also studied the influence of different curing systems containing Activ8 on the cure characteristics and performance of SBR composites filled with carbon black or nanosized silica. The influence of the filler on the activity and efficiency of the curing system is also discussed. We believed that Activ8 can accelerate curing reactions due to the presence of dibenzyldithiocarbamate salt and can interact with the filler, especially silica, as a result of proper functionalization. Thus, Activ8 can contribute to reducing the adsorption of the curatives on the filler surface. This is especially important in the case of elastomer composites filled with nanosized silica, which due to its large specific surface area has a high ability to adsorb the curing system. This significantly reduces the crosslink density of the vulcanizates and consequently deteriorates their performance.

The accelerating activity of Activ8 was previously reported by Bosch 2020 for the sulfur vulcanization of NR compounds filled with carbon black and SBR compounds with high silica loading [13]. However, the performance of Activ8 was studied in combination with only sulfenamide accelerators, so not with MBT and DPG. As it is known from the literature reports [9,10,11,12], depending on the type of vulcanization accelerators used at the same time, their interaction may be different and the synergistic effect leading to the improvement of curing characteristics of rubber compounds and the performance of vulcanizates is not always observed. Therefore, it seems reasonable to evaluate the effect of Activ8 in combination with the accelerators of different characteristics than sulfenamides, i.e., from the group of thiazoles and guanidines. Additionally, contrary to Bosch’s 2020 study, the silica-filled rubber compounds did not contain any silane, because we intended to investigate whether Activ8, due to the content of properly functionalized poly(ethylene oxide) (PEO) terminated with silicate compound, actually interacts with the surface of nanosized silica and reduces its ability to adsorb the curing system.

## 2. Materials and Methods

### 2.1. Materials

**Scheme 1:**
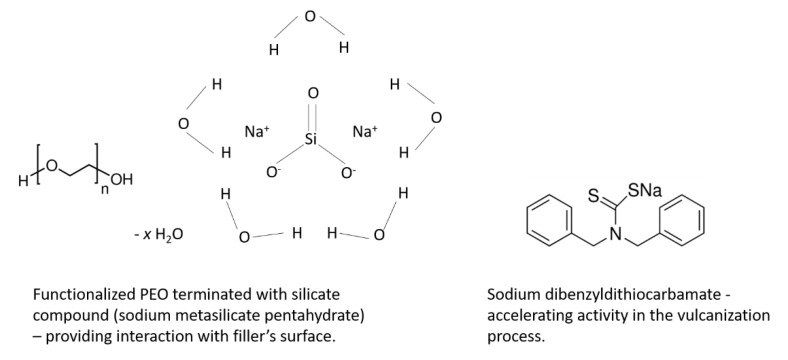
Structures of main active ingredients of Activ8.

SBR (KER1500) rubber with 23.5% bonded styrene and Mooney viscosity ML1 + 4 (100 °C): 50 was provided by Synthos SA (Oswiecim, Poland). It was crosslinked with sulfur in the presence of 2-mercaptobenzothiazole (MBT) or 1,3-diphenylguanidine (DPG) as accelerators (Sigma-Aldrich, Poznan, Poland). Zinc oxide characterized with a specific surface area of 10 m^2^/g (ZnO; Huta Bedzin, Będzin, Poland), together with stearic acid (St.A.; Sigma-Aldrich, Poznan, Poland), were applied as standard activators. As an additional activator/accelerator of sulfur vulcanization, Activ8 was used in the form of Premix Acti8 containing 50% active substance (provided by Torimex-Chemicals, Konstantynów Łódzki, Poland) mixed with silica and predispersed on a poly(ethylene oxide) (PEO) carrier. Activ8 material is a functionalized PEO terminated with silicate compound to provide its interaction with fillers. In addition, Activ8 contains sodium dibenzyldithiocarbamate, which ensures its accelerating activity in the vulcanization process [13]. Carbon black N550, provided by Konimpex Chemicals (Konin, Poland), and fumed silica Aerosil 380 (Evonik Industries, Essen, Germany) were applied as fillers. The structures of two main active ingredients of Activ8 are presented in Scheme 1.

### 2.2. Preparation and Characterization of the SBR Rubber Compounds and Vulcanizates

A laboratory two-roll mill was employed to prepare rubber compounds with the formulations specified in Table 1 and Table 2. A two-step procedure was applied during rubber compounds manufacturing. First, two masterbatches were prepared containing the sulfur, the zinc oxide, the stearic acid, and the proper filler. Then, the masterbatches were divided into equal pieces, and the vulcanization accelerator (MBT or DPG) was introduced into each of these pieces, together with the Activ8, with the exception of the reference rubber compounds, where only ZnO and St.A. were used as activators.

The SBR rubber compounds were vulcanized at 160 °C with an up to 90% increase in torque. The optimal vulcanization time (t_90_) was determined following the procedure described in the standard PN-ISO 6502:2007 using the MDR 2000 Moving Die Rheometer produced by Alpha Technologies (Heilbronn, Germany).

A differential scanning calorimeter (DSC1) (Mettler Toledo, Greifensee, Switzerland) was used to identify the range of SBR vulcanization temperatures and the energetic effect (enthalpy) of this process. Small pieces of rubber compounds with a mass of approximately 10 mg were hermetically sealed in aluminum crucibles and placed in the analyzer cell. After starting the measurement, samples were heated from −100 to 250 °C in an inert atmosphere (argon, 20 mL/min) at a heating rate of 10 °C/min.

The crosslink density of the SBR vulcanizates was investigated according to the PN-74/C-04236 standard procedure by their equilibrium swelling in toluene, and calculated based on the Flory–Rehner equation [14]. The Huggins parameter relating to interaction of the elastomer with a proper solvent (toluene) was defined using Equation (1) [15], where *V_r_* is the elastomer’s volume fraction in the swollen gel.

*µ* = 0.37 + 0.56 *V_r_*. (1)

The mechanical performance of the SBR vulcanizates during tension were investigated using the ISO-37 [16] with a ZWICK Roell 1435 (Zwick Roell, Ulm, Germany) testing machine. Five dumb-bell samples (test length of 20 mm and width of 4 mm) from each vulcanizate were examined and the mean value of the determinations was taken as the result.

The Shore A hardness was measured for disc-shaped samples of the SBR vulcanizates using a microcomputer-controlled hardness tester, ZwickRoell 3105 (Zwick/Roell, Ulm, Germany), according to the ISO-868 [17].

A DMA/SDTA861e (Mettler Toledo, Greifensee, Switzerland) analyzer was applied to examine the viscoelastic behavior of the SBR vulcanizates as a function of temperature. Measurements of the dynamic moduli were carried out in a tension mode over the temperature range of −100 to 80 °C with a heating rate of 3 °C/min. Samples of the vulcanizates in the shape of cuboids with length of 10.5 mm, width of 4 mm and thickness of 1 mm were stretched via oscillation with a frequency of 1 Hz and a strain amplitude of 4 µm. The temperature of the SBR glass transition (T_g_) was derived from the maximum of tan δ = f(T) plot, where tan δ is the loss factor and T is the measurement temperature.

The thermo-oxidative aging of the vulcanizates was conducted at a temperature of 100 °C for 7 days. To evaluate the resistance of the SBR elastomers to aging, their mechanical performance at static conditions and crosslink densities after aging were investigated and compared to the data obtained for the non-aged vulcanizates. Finally, the aging factor (AF) was determined following the procedure described in [18].

The thermal stability of the vulcanizates was investigated using a thermogravimetric TGA/DSC1 (Mettler Toledo, Greifensee, Switzerland) analyzer for small samples of the vulcanizates with a mass of approximately 10 mg. The measurement program consisted of two segments: (1) Heating 25–600 °C, argon, 50 mL/min, heating rate 20 °C/min; (2) heating 600–900 °C, air, 50 mL/min, heating rate 20 °C/min.

## 3. Results and Discussion

### 3.1. Effect of the Curing Systems on the Cure Characteristics of the SBR Compounds and the Crosslink Densities of the Vulcanizates

The influence of the curing system (i.e., the type of primary accelerator used), the addition of Activ8, and the content of ZnO on the cure characteristics of the SBR rubber compounds was estimated based on rheometer measurements at 160 °C. The characteristic parameters of the SBR compound vulcanization and the crosslink densities of the vulcanizates are provided in Table 3.

As mentioned, the tested rubber compounds contain MBT or DPG as the primary accelerator, ZnO as the vulcanization activator, and Activ8 as an additional activator. However, due to the presence of dibenzyldithiocarbamate salt, Activ8 is supposed to act as a vulcanization accelerator as well. To find out whether Activ8 allowed to reduce the amount of ZnO in the SBR, some of the rubber compounds contained three parts per hundred of rubber (phr) of ZnO instead of 5 phr and increased to 2 phr of Activ8.

The minimum torque (S_min_) during the rheometric measurements corresponded to the viscosity of the uncured rubber compounds. Therefore, it can be concluded that the type of curing system had no influence on the viscosity of the uncured SBR compounds filled with CB. Similarly, Activ8 and reducing the content of ZnO did not affect the viscosity of the uncured SBR compounds filled with CB compared to the minimum torque of the reference SBR compound containing 5 phr of ZnO as an activator. As expected, the uncured silica-filled rubber compounds exhibited considerably higher viscosity, and thus higher S_min_ than the SBR compounds filled with CB. This resulted from the application of fumed nanosized silica with quite large specific surface area, which increased the viscosity and the stiffness of the rubber compounds compared to carbon black N550. It should be noted that the uncured SBR compounds containing Activ8 showed an approximately 1.7 dNm lower S_min_ and, thus, lower viscosity than the reference sample with ZnO. This was likely due to the plasticizing effect of Activ8, which contains a functionalized poly(ethylene oxide) (POE).

The torque increment (ΔS) during the rheometric measurements corresponded to the degree of crosslinking of the elastomer. Applying Activ8 had no considerable influence on the torque increment of the CB-filled SBR compounds containing the MBT accelerator. The differences between ΔS values for these rubber compounds were within the range of standard deviation. Referring to the CB-filled SBR compounds containing the DPG accelerator, no effect of Activ8 on ΔS was observed for the rubber compound with 5 phr of ZnO. On the other hand, the rubber compound with a reduced amount of ZnO exhibited a 2.8 dNm lower torque increment than the reference sample, despite the increased amount of Activ8. In the case of the silica-filled rubber compounds cured with DPG, Activ8 did not affect the torque increment during vulcanization.

As is commonly known, MBT provides a medium cure rate, whereas DPG results in a rather slow cure rate but is safe in terms of rubber compound processing. Therefore, the SBR compounds with MBT exhibited a considerably shorter optimal vulcanization time than DPG-containing SBR. Applying Activ8 resulted in a considerably higher cure rate compared to the conventional ZnO/St.A. system. This was due to the accelerating action of the dibenzyldithiocarbamate salt, which is present in Activ8. As a consequence, the rubber compounds with Activ8 exhibited a significantly shorter optimal vulcanization time (t_90_) than the SBR cured without this ingredient. The most pronounced accelerating effect of Activ8 was obtained for the DPG-containing SBR compounds, especially those filled with nanosized silica. Applying Activ8 in the CB-filled rubber compounds containing DPG reduced the optimal vulcanization time from 32 min for the reference sample to 15 min for the SBR compounds containing 2 phr of Activ8, despite the 40% lower content of ZnO. In contrast, the reference rubber compound filled with silica showed an optimal vulcanization time of 53 min, which is approximately 20 min longer than the SBR compounds containing CB as a filler. This was likely due to the adsorption of the curing system, especially DPG, on the expanded surface of the nanosized silica [19]. Most importantly, Activ8 allowed to shorten the t_90_ of the silica-filled SBR compounds to 15 min and 8 min for 1 phr or 2 phr of Activ8, respectively. Moreover, this significant reduction in t_90_ was possible even the amount of ZnO was 40% lower compared to the reference rubber compound. We believe that the positive influence of Activ8 on the cure rate of the silica-filled SBR resulted not only from the accelerating action of the dibenzyldithiocarbamate salt, but may also be due to the interaction of Activ8 with the filler surface.

It should be noted that Activ8 is a physical mixture of two active ingredients, such as functionalized POE terminated with a silicate compound and sodium dibenzyldithiocarbamate. Therefore, when considering the action of Activ8 in rubber compounds, it should be expected that functionalized with a silicate compound POE will interact with the surface groups of fillers (e.g., silanol groups of silica), and, consequently, decrease the ability of the nanosized silica surface to adsorb DPG, thereby improving the efficiency of using the DPG accelerator. On the other hand, sodium dibenzyldithiocarbamate will participate in crosslinking reactions according to the mechanism typical for dithiocarbamate accelerators. Such a mechanism has been described in detail by Nieuwenhuizen et al., 1997 [20] and confirmed by further studies [21].

As expected, the reference rubber compound cured with DPG as an accelerator exhibited significantly longer scorch time than the MBT-containing SBR compounds. Owing to the accelerating effect, Activ8 considerably reduced the scorch time of the rubber compounds, which is unfavorable for processing safety. However, the scorch times were determined at 160 °C, whereas rubber compounds can be formed and processed at lower temperatures, e.g., 100–125 °C. It is known that the cure rate decreases as the temperature decreases. Therefore, lowering the processing temperature of rubber compounds should result in longer scorch times and improved processing safety [22].

Analyzing the data compiled in Table 3, we observed that the curing system had an impact on the crosslink density of the SBR compounds. The CB-filled vulcanizates cured with MBT showed higher crosslink densities compared to those containing the DPG accelerator. The addition of Activ8 affected the crosslink density of the SBR vulcanizates. The influence of Activ8 on the number of crosslinks in the elastomer network depends on the type of accelerator and the filler used. Applying Activ8 increased the crosslink density of the CB-filled vulcanizates cured with MBT, but had no effect on the crosslink density of the SBR composites containing the DPG accelerator. As expected, the most pronounced influence of Activ8 was obtained for the SBR vulcanizates filled with nanosized silica, which exhibited significantly higher crosslink densities compared to the reference vulcanizate with ZnO. This may have resulted from the reduction in the ability of the silica surface to adsorb the curing system, including sulfur. Most importantly, Activ8 allowed for reducing the content of ZnO in the SBR composites by 40% without a deterioration of their crosslink density.

In the next step of this work, the impact of the curing system and Activ8 on the temperature and enthalpy of the vulcanization was established based on the results of DSC analysis. The data for SBR compounds filled with CB or silica are summarized in Table 4.

The data presented in Table 4 show that the curing system affects the range of the vulcanization temperature of the SBR compounds, as well as the enthalpy of this process. The vulcanization of the rubber compounds containing MBT as the primary accelerator began at a temperature approximately 20–30 °C lower compared to the SBR cured in the presence of the DPG accelerator. The influence of Activ8 on the temperature and enthalpy of the vulcanization depends on the primary accelerator applied and the filler. As far as the CB-filled rubber compounds cured with MBT are concerned, Activ8 reduced the onset vulcanization temperature by 10–14 °C compared to the SBR composite containing the conventional ZnO/St.A. system. Consequently, the temperature of the vulcanization peak was reduced by 9–15 °C. Activ8 did not affect the enthalpy of the vulcanization, since the values of ΔH_vul_ were within the range of standard deviation. A similar influence of Activ8 on the vulcanization temperature and enthalpy was obtained for the silica-filled SBR compounds containing DPG as the accelerator. In contrast, Activ8 had no influence on the vulcanization temperature of the CB-filled SBR cured with DPG, while it reduced the enthalpy of the vulcanization by almost twice in comparison with the rubber compound containing ZnO.

Most importantly, the application of Activ8 allowed for considerable reduction in the optimal vulcanization time and did not rise the range of the vulcanization temperature compared to the SBR cured in the presence of the ZnO/St.A. system.

### 3.2. Effect of the Curing Systems on the Mechanical Properties and Hardness of the SBR Vulcanizates

Having established the impact of the curing system and Activ8 on the curing characteristics of the SBR rubber compounds, we then investigated the tensile properties and hardness of the vulcanizates, the results of which are given in Table 5.

From the data collected in Table 5, it follows that the changes in the SE_300_ modulus of the SBR vulcanizates under the influence of the curing system, including Activ8, are consistent with the changes in the crosslink density. Therefore, the CB-filled vulcanizates cured with MBT exhibited the highest SE_300_ among those tested. Moreover, the SE_300_ modulus of the vulcanizates containing Activ8 was higher compared to that of the reference vulcanizate with 5 phr of ZnO. Activ8 did not affect the SE_300_ of the CB-filled SBR cured with DPG, whereas the vulcanizates filled with nanosized silica showed a significantly higher SE_300_ compared to the vulcanizate cured in the presence of the ZnO/St.A system.

The curing system seems to affect the tensile strength of the SBR vulcanizates. Most of the CB-filled vulcanizates containing DPG exhibited an approximately 4 MPa higher TS than those cured with MBT. Activ8 did not considerably affect the TS values of the CB-filled vulcanizates containing MBT, whereas their elongation at break (EB) was approximately 45% lower compared to the reference vulcanizate without Activ8 due to the higher crosslink density. A similar effect of Activ8 on the mechanical performance was obtained for the CB-filled vulcanizate cured with DPG and containing 1 phr of Activ8 and 5 phr of ZnO. However, the vulcanizate containing Activ8 and a 40% lower amount of ZnO (3ZnO/2Activ8) exhibited an approximately 7 MPa lower TS compared to the reference vulcanizate and the one containing Activ8 and 5 phr of ZnO.

Applying Activ8 considerably increased the TS and reduced the EB of the silica-filled vulcanizates compared to those obtained using the conventional ZnO/St.A. system. This resulted from the considerable increase in the crosslink density of the vulcanizates. It is worth noting that the vulcanizates containing Activ8 demonstrated a 2–3 times higher TS and an approximately 150% lower EB than the reference composite cured in the presence of the ZnO/St.A activator.

As far as the hardness of the vulcanizates is concerned, using Activ8 increased the hardness of the CB-filled vulcanizates cured with MBT and the silica-filled vulcanizates containing DPG. This resulted from the increase in the crosslink density of the vulcanizates compared to that of the reference samples without Activ8. Since Activ8 had no significant effect on the crosslink density of the MBT-containing vulcanizates, no significant changes in the hardness of these composites were observed either.

**Figure 1:**
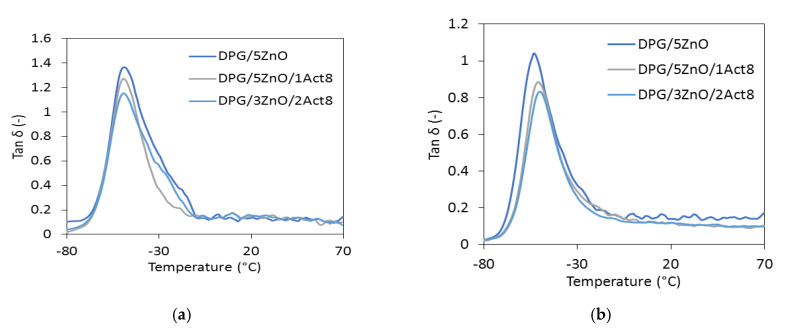
The mechanical loss factor (tan δ) versus the temperature of the DPG-containing SBR vulcanizates filled with: (**a**) CB; (**b**) nanosized silica.

The mechanical performance of SBR elastomers were also tested under dynamic conditions. Thus, the influence of the curing system, the addition of Activ8, and the reduction in zinc oxide on the mechanical loss factor (tan δ) was evaluated with the dynamic mechanical analysis (DMA). Plots of tan δ versus the temperature for the elastomers containing DPG as the accelerator are presented in Figure 1, and the data are collected in Table 6.

The transition of the elastomer from a glassy to a rubbery elastic state can be seen in the DMA curves of the SBR vulcanizates as the peak of tan δ plot versus temperature. A maximum of this peak represents the glass transition temperature (T_g_). The determined T_g_ for the reference vulcanizates filled with CB was approximately −48 °C, regardless of the accelerator used, whereas for the vulcanizate with nanosized silica, the T_g_ value was −51 °C. Thus, it was concluded that the type of vulcanization accelerator and the addition of Activ8 did not considerably influence the T_g_ of the SBR composites. On the other hand, the type of the filler and the application of Activ8 had a significant influence on the tan δ determined at T_g_. The effect of the fillers on tan δ resulted from the different stiffnesses of the vulcanizates. The CB-filled SBR composites were less stiff and thus more elastic than the vulcanizates filled with nanosized silica, since this filler considerably stiffens vulcanizates. Therefore, the CB-filled vulcanizates exhibited significantly higher tan δ at T_g_ compared to the silica-filled SBR. Regardless of the filler and the vulcanization accelerator used, the vulcanizates containing Activ8 exhibited a lower tan δ at T_g_ than the reference SBR composites cured with the ZnO/St.A. system. The decrease in tan δ was due to the increased crosslink density of the vulcanizates. An elastomeric network with a high concentration of crosslinks restricts the mobility of the elastomer chains, as well as decreases the elasticity of the vulcanizates.

In contrast, the type of filler and the addition of Activ8 had no considerable influence on the values of tan δ after the elastomer transitioned to the elastic state. The tan δ curves presented in Figure 1 for most of the vulcanizates almost overlap in the rubbery elastic region, whereas the tan δ values presented in Table 6 in the range of 25–60 °C are comparable, considering the measurement uncertainty.

The mechanical loss factor is considered as a measure of an elastomer’s capacity to supress vibration. The curing system and Activ8 did not significantly influence the tan δ at temperatures above 25 °C, so the damping properties of the SBR vulcanizates were in the rubbery elastic state. The mechanical loss factors of the SBR vulcanizates in the temperature range of 25–60 °C were comparable regarding the experimental error. Moreover, the SBR vulcanizates demonstrated stable dynamic mechanical behavior in the rubbery elastic region, because the values of the mechanical loss factor did not fluctuate significantly as the function of temperature after the elastomer transitioned to the elastic state.

**Figure 2:**
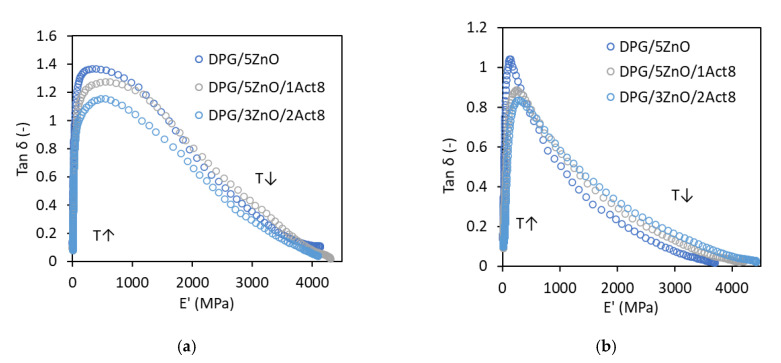
Tan δ versus the storage modulus E’ of the DPG-containing SBR vulcanizates filled with: (**a**) CB; (**b**) nanosized silica.

To examine the effect of the curing system on the thermorheological behavior of SBR composites, plots of the tan δ versus the storage modulus E’ (wicket-plots) were prepared (Figure 2). This method was commonly used to investigate whether the elastomer demonstrates a behavior of thermorheologically simple or complex material [23,24]. According to Çakmak et al. (2014), material is characterized with thermorheologically simple behavior if the loss modulus or tan δ are a unique function of E’ over all temperatures and frequencies tested [23]. Thus, SBR composites studied seem to demonstrate a thermorheologically simple material behavior. The relation between tan δ and E’ is of the same shape regardless of the presence and the content of Activ8 in the curing system. It is worth noting that the type of filler used influences the shape of the tan δ versus E’ curves, which are narrower for a material filled with nanosized silica compared to vulcanizates with CB showing lower hardness and stiffness. A similar relation between the shape of tan δ versus E’ curves and hardness of the material was observed for poly(norbornene) composites [25].

### 3.3. Effect of the Curing Systems on the Resistance of the SBR Vulcanizates to Thermo-Oxidative Aging

**Figure 3:**
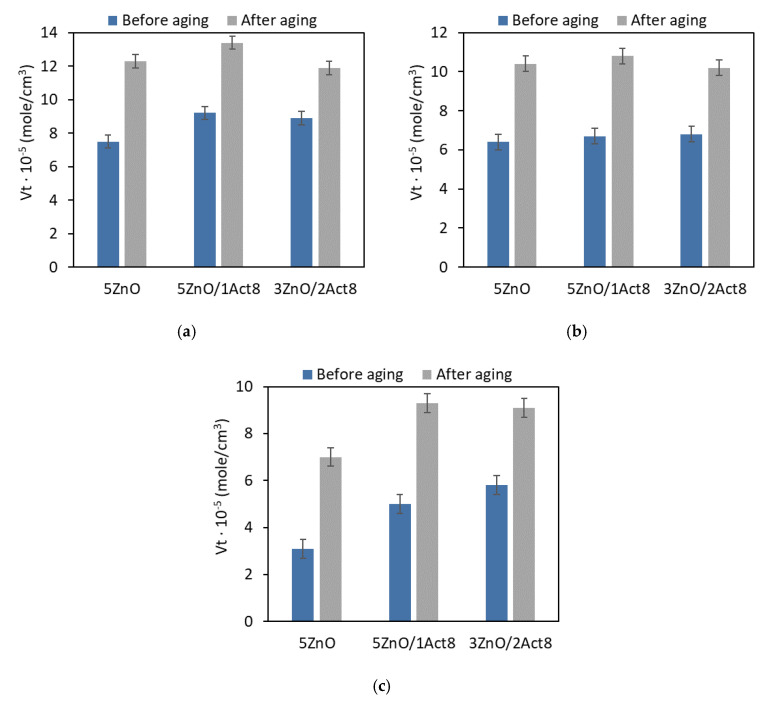
Effect of thermo-oxidative aging on the crosslink density of the SBR vulcanizates containing: (**a**) CB and MBT; (**b**) CB and DPG; (**c**) silica and DPG.

**Figure 4:**
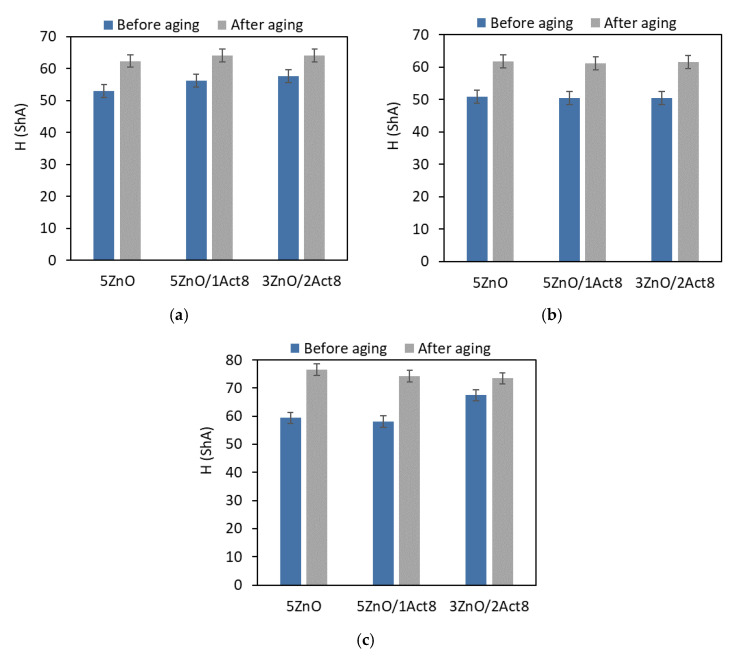
Effect of thermo-oxidative aging on the hardness of the SBR vulcanizates containing: (**a**) CB and MBT; (**b**) CB and DPG; (**c**) silica and DPG.

**Figure 5:**
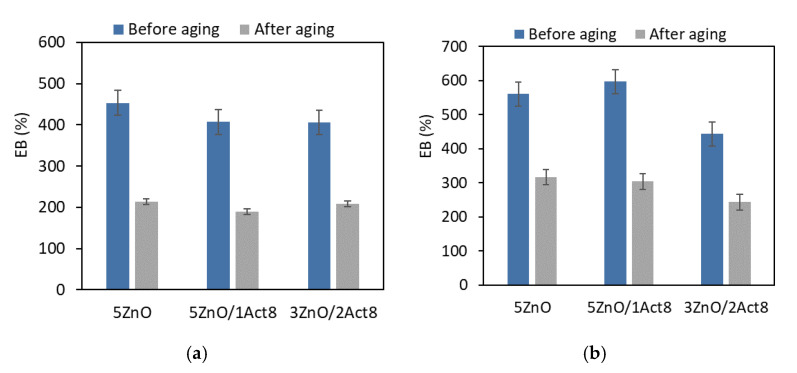

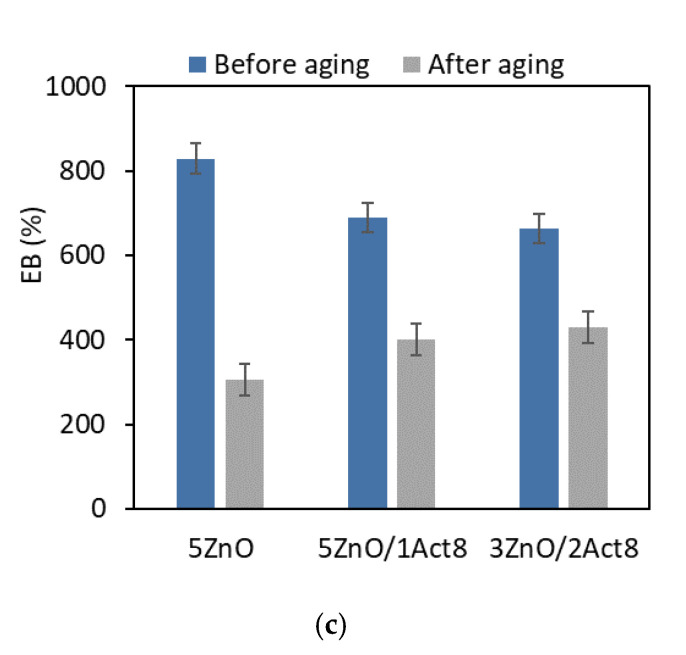
Effect of thermo-oxidative aging on the elongation at break of the SBR vulcanizates containing: (**a**) CB and MBT; (**b**) CB and DPG; (**c**) silica and DPG.

**Figure 6:**
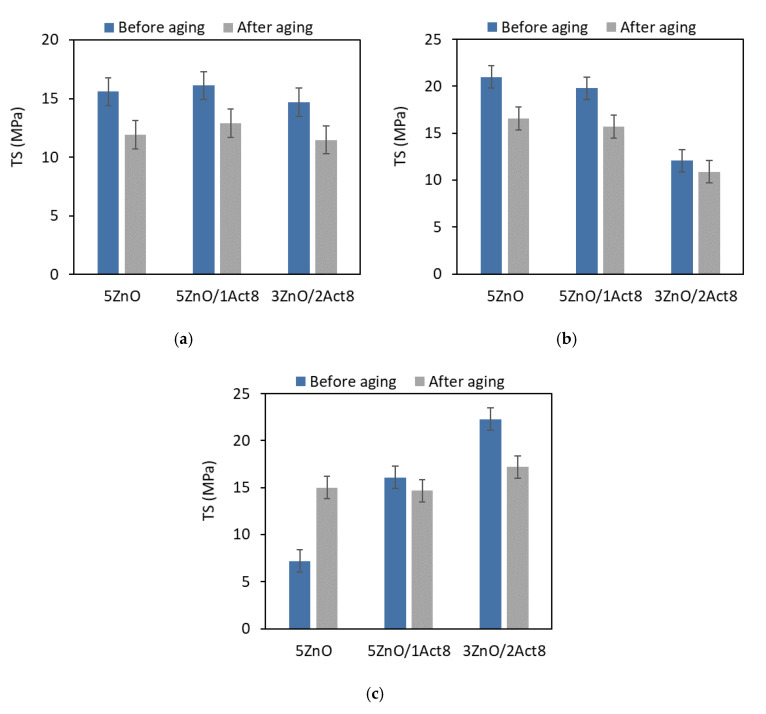
Effect of thermo-oxidative aging on the tensile strength of the SBR vulcanizates containing: (**a**) CB and MBT; (**b**) CB and DPG; (**c**) silica and DPG.

During their exploatation, SBR rubber products are subjected to unfavorable external factors. One of these factors is high temperature, which causes thermo-oxidative aging and, consequently, changes the material’s properties. Therefore, vulcanizates containing different curing systems were subjected to prolonged exposure to elevated temperature (100 °C) for seven days. The effect of the curing system and the addition of Activ8 on the vulcanizates’ resistance to thermo-oxidative aging was investigated through the change in their tensile properties, hardness, and crosslink density (Figure 3, Figure 4, Figure 5 and Figure 6).

When analyzing the crosslink density of the vulcanizates, it was concluded that prolonged exposure to 100 °C initiated further curing of the SBR composites (Figure 3). Vulcanization, like other technological processes, does not proceed with 100% efficiency. During vulcanization, the curatives contained in rubber compounds are used to an optimal degree, which does not mean that they react completely. Residues of the curing system, including sulfur, may still be present in the resulting rubber composite, which may cause further curing of the elastomer during prolonged exposure to elevated temperatures. The same effect of thermo-oxidative aging on the number of crosslinks in the elastomer network has been repeatedly mentioned for other polymers [26,27]. The highest increase in the crosslink density due to thermo-oxidative aging was achieved for the vulcanizates filled with nanosized silica, which showed the lowest crosslink density before the aging process. On the other hand, the lowest increase in the content of the crosslink in the elastomer network under the influence of aging occurred for the CB-filled vulcanizates containing MBT, that is, those with the highest crosslink density before the aging process. Most importantly, applying Activ8 did not considerably affect the changes in the crosslink density of the SBR composites due to the thermo-oxidative aging.

The impact of thermo-oxidative aging on the hardness of the SBR elastomers is shown in Figure 4.

The SBR vulcanizates exhibited higher hardness after the thermo-oxidative aging process compared to the non-aged samples. This resulted from the increase in their crosslink density. The smallest changes in hardness were obtained for the CB-filled vulcanizates containing MBT, whose hardness after aging increased by 8–11 ShA. It should be noted that these elastomers showed the smallest increase in crosslink density due to the aging process. The highest hardness (74–80 ShA) after thermo-oxidative aging was exhibited for the vulcanizates filled with nanosized silica, for which the most pronounced increase in crosslink density was observed. The hardness increased by 14 ShA for the vulcanizates with Activ8 and by 20 ShA for the reference sample with the ZnO/St.A. system. Activ8 did not contribute to a greater change in the hardness of the vulcanizates due to aging compared to the reference SBR composites without this ingredient.

Another result of the increased crosslink density under the influence of thermo-oxidative aging was a significant reduction in the EB of the vulcanizates (Figure 5).

Regardless of the filler type or the vulcanization accelerator and activator, most of the vulcanizates demonstrated an elongation at break after the aging process of approximately 250% lower compared to the EB of the non-aged vulcanizates. The highest EB after thermo-oxidative aging was observed for the SBR vulcanizates filled with nanosized silica (in the range of 400–540%).

As expected, due to the increase in the crosslink density, thermo-oxidative aging affected the TS of the vulcanizates (Figure 6).

It is commonly known, that TS increases with an increase in the crosslink density up to a critical concentration of crosslinks in the elastomer network. A further increase in the crosslink density results in the deterioration of the TS, since the over-cured vulcanizate becomes more brittle and prone to rupture under external stress. Therefore, thermo-oxidative aging reduced the TS of the vulcanizates, except for the reference sample filled with nanosized silica, for which the TS increased by approximately 8 MPa compared to the non-aged vulcanizate. It should be noted that this vulcanizate exhibited a much lower crosslink density than the other CB-filled or silica-filled vulcanizates. Thus, the TS of this vulcanizate increased with the an increase in the crosslink density due to the aging process.

In order to quantify the resistance to thermo-oxidative aging and to facilitate a comparison of the results obtained for the tested vulcanizates, the AF was calculated based on the changes in the vulcanizates’ TS and EB resulting the aging process (Table 7). The higher the AF coefficient, the smaller the changes in the TS and EB of the vulcanizate due to aging and, consequently, the greater the resistance to thermo-oxidative aging.

As was mentioned, thermo-oxidative aging at 100 °C increased the crosslink density of the SBR vulcanizates and, consequently, considerably reduced their tensile strength and elongation at break. Therefore, their AF coefficients were in the range of 0.36–0.52. Only the reference vulcanizates filled with nanosized silica were characterized by an AF of 1.44 due to the significant increase of its tensile strength. However, the tensile strength improvement resulted from the further curing of the sample, which, before the aging process, had a much lower crosslink density than the other vulcanizates. Therefore, the AF coefficient of higher than 1 determined for this vulcanizate should not be considered as a confirmation of its high aging resistance. Most importantly, Activ8 did not considerably affect the AF of the CB-filled vulcanizates, that is, it did not significantly impact their resistance to thermo-oxidative aging. The silica-filled SBR vulcanizates containing Activ8 exhibited an AF of approximately 0.5, which is lower than the reference sample cured with ZnO/St.A., but comparable to that of the CB-filled vulcanizates.

### 3.4. Effect of the Curing Systems on the Thermal Stability of the SBR Vulcanizates

**Figure 7:**
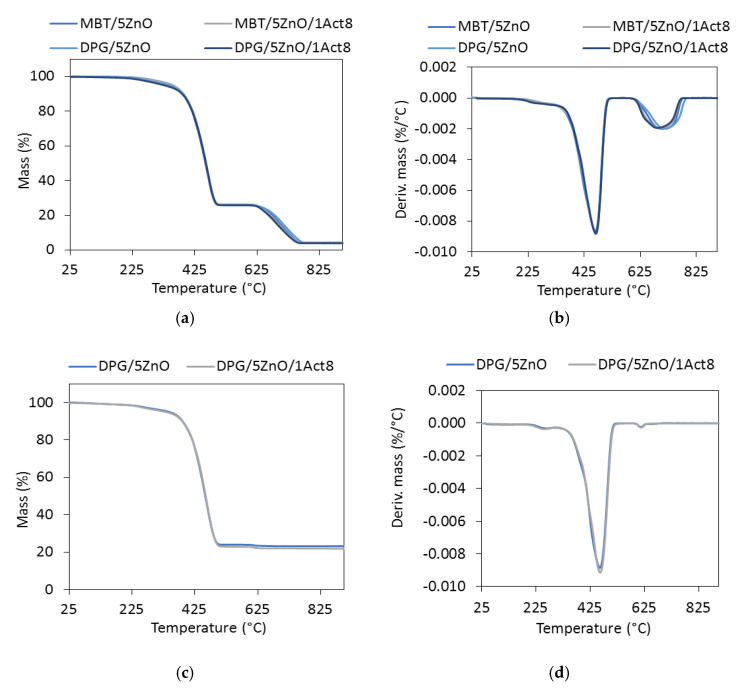
The thermogravimetric (TG) and differential thermogravimetric (DTG) curves of the SBR vulcanizates containing: (**a**,**b**) CB; (**c**,**d**) silica.

Having examined the the resistance of the SBR composites to thermo-oxidative aging, we than performed thermogravimetric (TG) analysis to determine the effect of primary accelerator (DPG or MBT), Activ8 and reduction of ZnO level on the thermal stability of the vulcanizates. The results are summarized in Table 8, while the TG and differential thermogravimetric (DTG) curves for the reference vulcanizates and those containing 1 phr of Activ8 are presented in Figure 7.

**Figure 8:**
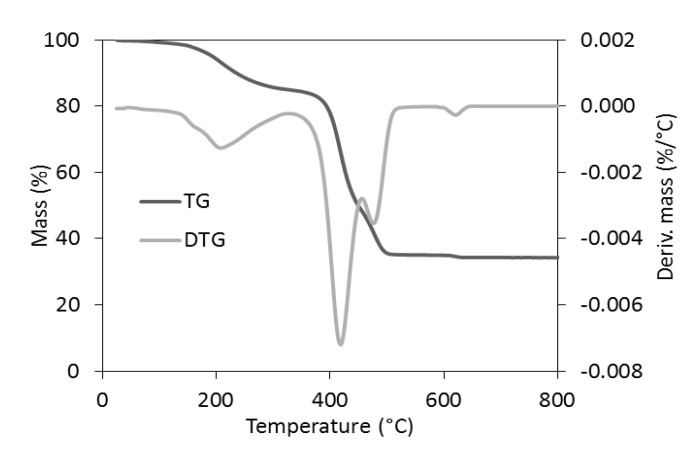
The TG and DTG curves of Premix Acti8.

The TG analysis revealed that the reference samples had different thermal stability depending on the curing system and the addition of Activ8 (Table 8). Considering T_5%_, i.e., the decomposition temperature at a 5% mass change, as the onset decomposition temperature, the highest thermal stability was exhibited by the CB-filled vulcanizates containing MBT (T_5%_ in the range of 353–342 °C), whereas the lowest decomposition temperatures were determined for the CB-filled SBR cured with DPG (T_5%_ in the range of 319–337 °C). Regardless of the curing system used, applying Activ8 deteriorated the thermal stability of the SBR composites. Moreover, the T_5%_ temperature of the vulcanizate decreased by approximately 6 °C, when the content of Activ8 in the rubber composite increased from 1 to 2 phr. Therefore, it was concluded that the reduction in the onset decomposition temperature of the SBR vulcanizates resulted from the thermal decomposition of Activ8, which preceded the thermal decomposition of the rubber and the other ingredients of the tested composites. Low thermal stability of the Activ8 was confirmed by TG analysis. The results are given in Figure 8 and Table 9. The measurements were conducted for the commercial product Premix Acti8, which contains 50% Activ8.

Analysis of the TG and DTG curves revealed that the thermal decomposition of Premix Activ8 proceeded in several stages. This is because Premix Acti8 is a blend of Activ8 with silica and POE carriers. Furthermore, Activ8 itself consists of POE functionalized with silicate compound and additionally contains dibenzyldithiocarbamate salt. The first step of the thermal decomposition of Premix Acti8 occurred in the temperature range of 80–330 °C with a DTG peak temperature of 217 °C. The mass loss of the sample at this stage of thermal decomposition was approximately 15.3%, which was likely due to the moisture desorption from the silica accompanied by the thermal decomposition of the dibenzyldithiocarbamate salt [28]. The main step of the thermal decomposition of Premix Acti8 proceeded in the temperature range of 330–600 °C with a mass loss of 49.5%, which may have resulted mainly from the thermal decomposition of POE [29]. At temperatures above 600 °C, after changing the gas into air, the remains of the thermal decomposition were burnt, leaving a mineral residue of approximately 34.5% resulting from the presence of silica. It should be noted that the T_5%_ of Activ8 was approximately 193 °C, which is much lower than that of the SBR vulcanizates and the pure SBR [30]. Thus, it was confirmed that the worse thermal stability of the Activ8-containing SBR vulcanizates compared to the vulcanizates without this additive may be due to the low thermal stability of Activ8 itself. However, it should be noted that the SBR vulcanizates containing Activ8 were thermally stable up to a temperature of approximately 320 °C, which is important for their potential technological applications.

## 4. Conclusions

The composition of the curing system, such as the type of primary accelerator (DPG or MBT) and the application of Activ8 as an additional activator/accelerator of vulcanization, was confirmed to affect the activity of the curing system in the sulfur vulcanization of SBR. Furthermore, the activity of the curing system depended on the nature of the filler used.

As expected, the SBR compounds with MBT exhibited a considerably shorter optimal vulcanization time than the DPG-containing SBR. Most importantly, applying Activ8 resulted in a considerably higher cure rate compared to the conventional ZnO/St.A. system, which was due to the accelerating action of the dibenzyldithiocarbamate salt contained in Activ8. The most pronounced accelerating effect of Activ8 was observed for the DPG-containing SBR compounds, filled with nanosized silica, although the amount of ZnO was 40% lower (3 phr) compared to the reference rubber compound (5 phr). The positive influence of Activ8 on the cure rate of the silica-filled SBR may also have resulted from the interaction of Activ8 with the filler surface, since Activ8 consists of functionalized POE terminated with a silicate compound, allowing interaction with the silica surface and, consequently, decreasing the ability of the nanosized silica surface to adsorb DPG.

The curing system had influence on the crosslink density of the SBR. The CB-filled vulcanizates cured with MBT exhibited higher crosslink densities compared to those cured with DPG. The influence of Activ8 on the crosslink density depended on the type of accelerator and the filler used. Activ8 increased the crosslink density of the CB-filled vulcanizates cured with MBT, but had no influence on the crosslink density of the SBR composites containing DPG. The most pronounced influence of Activ8 was obtained for the SBR filled with nanosized silica, which exhibited a significantly higher crosslink density compared to the reference vulcanizate with the ZnO/St.A. system.

The curing system seemed to affect the mechanical properties of the SBR vulcanizates. Most of the CB-filled vulcanizates containing DPG exhibited higher TS than those cured with MBT. Activ8 had not considerable impact on the tensile strength of the CB-filled vulcanizates containing MBT, whereas it considerably enhanced the tensile strength and reduced the elongation at break of the silica-filled vulcanizates compared to using the conventional ZnO/St.A. activator. This was due to the considerable increase in the crosslink density of the elastomers.

Regardless of the filler and curing system used, the vulcanizates containing Activ8 showed lower tan δ at T_g_ than the reference SBR composites cured with the ZnO/St.A. system due to the increased crosslink density.

The curing system did not have a significant influence on the aging resistance of the SBR filled with CB. The silica-filled SBR vulcanizates containing Activ8 exhibited worse aging resistance than the reference sample cured with ZnO, but was comparable to the CB-filled vulcanizates.

The SBR cured with MBT accelerator exhibited slightly higher thermal stability than the DPG-containing SBR, especially when filled with CB. Regardless of the curing system used, the application of Activ8 deteriorated the thermal stability of the SBR composites, which was due to the low thermal stability of Activ8. However, the vulcanizates obtained were thermally stable up to a temperature of approximately 320 °C, which is satisfactory for their potential applications.

## Figures and Tables

**Table 1:** General formulation of the SBR rubber compounds with the MBT accelerator, parts per hundred of rubber (phr).

Ingredient	Reference Sample R1	Rubber Compounds with Activ8 SBR1–2	
SBR	100	100	100
Sulfur	2	2	2
MBT	2	2	2
ZnO	5	5	3
St.A.	1	1	1
CB	30	30	30
Activ8	-	1	2

SBR, styrene–butadiene elastomer; MBT, 2-mercaptobenzothiazole; ZnO, zinc oxide; St.A., stearic acid; CB, carbon black.

**Table 2:** General composition of the SBR rubber compounds with the DPG accelerator, phr.

Ingredient	Reference Samples R2 and R3	Rubber Compounds with Activ8 SBR3–6	
SBR	100	100	100
Sulfur	2	2	2
DPG	2.5	2.5	2.5
ZnO	5	5	3
St.A.	1	1	1
Filler *	30	30	30
Activ8	-	1	2

* Alternatively CB or silica. DPG, 1,3-diphenylguanidine.

**Table 3:** The cure characteristics of the SBR compounds at 160 °C and the crosslink density of the vulcanizates.

Activator	S_min_ (dNm)	ΔS (dNm)	t_02_ (min)	t_90_ (min)	ν_t_·10^−5^ (mole/cm^3^)
Rubber compounds with CB and MBT					
5ZnO	1.2	12.5	0.8	6	7.6
5ZnO/1Activ8	1.2	13.9	0.4	3	9.2
3ZnO/2Activ8	1.2	12.7	0.4	4	8.9
Rubber compounds with CB and DPG					
5ZnO	1.1	12.4	4.4	32	6.4
5ZnO/1Activ8	1.1	11.7	2.0	19	6.7
3ZnO/2Activ8	1.1	9.6	1.8	15	6.8
Rubber compounds with silica and DPG					
5ZnO	7.1	20.0	4.8	53	3.1
5ZnO/1Activ8	5.4	19.9	1.1	15	5.0
3ZnO/2Activ8	5.6	20.2	0.5	8	5.8

S_min_, minimum torque; ΔS, increment of torque during vulcanization; t_02_, scorch time; t_90_, optimal vulcanization time. Standard deviations: S_min_ ± 0.5, ∆S ± 1.7 dNm, t_02_ ± 0.2 min, t_90_ ± 0.9 min, and ν_t_ ± 0.4 × 10^−5^ mole/cm^3^.

**Table 4:** Temperature and enthalpy (ΔH_vul_) of SBR vulcanization determined by DSC.

Activator	Temperature of Vulcanization (°C)	Temperature of Peak (°C)	ΔH_vul_ (J/g)
Rubber compounds with CB and MBT			
5ZnO	158–203	178	4.1
5ZnO/1Activ8	144–184	163	3.2
3ZnO/2Activ8	148–192	169	3.5
Rubber compounds with CB and DPG			
5ZnO	171–241	214	9.2
5ZnO/1Activ8	173–245	215	4.8
3ZnO/2Activ8	172–242	214	5.0
Rubber compounds with silica and DPG			
5ZnO	182–238	209	13.8
5ZnO/1Activ8	175–238	203	12.3
3ZnO/2Activ8	165–231	198	12.0

Standard deviations: temperature ± 2 °C and ∆H ± 1.7 J/g. DSC, differential scanning calorimetry.

**Table 5:** Tensile properties and hardness of the SBR vulcanizates.

Activator	SE_300_ (MPa)	TS (MPa)	EB (%)	H (ShA)
Vulcanizates with CB and MBT				
5ZnO	8.8	15.6	453	51
5ZnO/1Activ8	10.9	16.1	407	56
3ZnO/2Activ8	9.9	14.7	406	55
Vulcanizates with CB and DPG				
5ZnO	7.2	21.0	561	49
5ZnO/1Activ8	6.8	20.3	573	48
3ZnO/2Activ8	7.0	13.6	534	47
Vulcanizates with silica and DPG				
5ZnO	1.4	7.2	829	57
5ZnO/1Activ8	2.6	16.1	690	60
3ZnO/2Activ8	3.6	22.3	664	66

SE_300_, modulus at a relative elongation of 300%; TS, tensile strength; EB, elongation at break; H, hardness. Standard deviations: SE_300_ ± 0.4 MPa, TS ± 1.2 MPa, EB ± 33%, and H ± 2 ShA.

**Table 6:** Glass transition temperature (T_g_) and mechanical loss factor (tan δ) of the SBR vulcanizates.

Activator	T_g_ (°C)	tan δ at T_g_ (−)	tan δ at 25 °C (−)	tan δ at 60 °C (−)
Vulcanizates with CB and MBT				
5ZnO	−48.0	1.39	0.11	0.09
5ZnO/1Activ8	−47.9	1.30	0.11	0.08
3ZnO/2Activ8	−48.1	1.27	0.12	0.10
Vulcanizates with CB and DPG				
5ZnO	−48.8	1.37	0.14	0.14
5ZnO/1Activ8	−49.1	1.27	0.14	0.11
3ZnO/2Activ8	−49.1	1.15	0.15	0.12
Vulcanizates with silica and DPG				
5ZnO	−51.0	1.04	0.14	0.14
5ZnO/1Activ8	−50.3	0.88	0.11	0.10
3ZnO/2Activ8	−49.2	0.83	0.12	0.10

Standard deviations: T_g_ ± 0.9 °C and tan δ ± 0.05.

**Table 7:** Aging factor (AF) of the SBR vulcanizates.

Activator	AF (−)
Vulcanizates with CB and MBT	
5ZnO	0.36
5ZnO/1Activ8	0.37
3ZnO/2Activ8	0.40
Vulcanizates with CB and DPG	
5ZnO	0.45
5ZnO/1Activ8	0.41
3ZnO/2Activ8	0.38
Vulcanizates with silica and DPG	
5ZnO	1.44
5ZnO/1Activ8	0.50
3ZnO/2Activ8	0.52

Standard deviation: AF ± 0.08.

**Table 8:** Onset temperature of thermal decomposition (T_5%_), temperature of the DTG peak (T_DTG_), and total mass loss (∆m) during the thermal decomposition of SBR vulcanizates.

Activator	T_5%_ (°C)	T_DTG_ (°C)	Δm 25–600 °C (%)	Δm 600–900 °C (%)	Residue at 900 °C (%)
Vulcanizates with CB and MBT					
5ZnO	353	482	74.0	22.1	3.9
5ZnO/1Activ8	349	483	73.6	22.0	4.4
3ZnO/2Activ8	342	480	74.5	22.1	3.4
Vulcanizates with CB and DPG					
5ZnO	337	483	74.2	22.0	3.8
5ZnO/1Activ8	325	483	73.9	22.0	4.1
3ZnO/2Activ8	319	481	74.6	22.1	3.3
Vulcanizates with silica and DPG					
5ZnO	342	478	76.0	0.8	23.2
5ZnO/1Activ8	332	478	77.0	0.9	22.1
3ZnO/2Activ8	327	476	77.2	0.8	22.0

Standard deviations: T_5%_ ± 1.3 °C, T_DTG_ ± 1.2 °C, and ∆m ± 1.3%.

**Table 9:** The onset decomposition temperature at a 5% (T_5%_) mass change, temperature of the DTG peak (T_DTG_), and total mass losses (∆m) during the thermal decomposition of Premix Acti8.

Parameter	
**T_5%_ (°C)**	193
**T_DTG(1st step)_ (°C)**	217
**T_DTG(2nd step)_ (°C)**	435
**Δm 80–330 °C (%)**	15.3
**Δm 330–600 °C (%)**	49.5
**Δm 600–800 °C (%)**	0.7
**Residue at 800 °C (%)**	34.5

Standard deviations: T_5%_ ± 1.5 °C, T_DTG_ ± 1.6 °C, and ∆m ± 1.1%.
